# Demonstration of a Transportable Fabry–Pérot Refractometer by a Ring-Type Comparison of Dead-Weight Pressure Balances at Four European National Metrology Institutes

**DOI:** 10.3390/s24010007

**Published:** 2023-12-19

**Authors:** Clayton Forssén, Isak Silander, Johan Zakrisson, Eynas Amer, David Szabo, Thomas Bock, André Kussike, Tom Rubin, Domenico Mari, Stefano Pasqualin, Zaccaria Silvestri, Djilali Bentouati, Ove Axner, Martin Zelan

**Affiliations:** 1 Department of Physics, Umeå University, SE-901 87 Umeå, Sweden; clayton.forssen@umu.se (C.F.); isak.silander@umu.se (I.S.); johan.zakrisson@umu.se (J.Z.); ove.axner@umu.se (O.A.); 2 Measurement Science and Technology, RISE Research Institutes of Sweden, SE-501 15 Borås, Sweden; eynas.amer@ri.se (E.A.); david.szabo@ri.se (D.S.); 3 Physikalisch-Technische Bundesanstalt (PTB), Abbestr 2-12, 10587 Berlin, Germanyandre.kussicke@ptb.de (A.K.); tom.rubin@ptb.de (T.R.); 4 Istituto Nazionale di Ricerca Metrologica (INRiM), Strada delle Cacce 91, 10135 Turin, Italy; d.mari@inrim.it (D.M.); s.pasqualin@inrim.it (S.P.); 5 Conservatoire National des Arts et Métiers, Laboratoire Commun de Métrologie (LNE-CNAM), 1 Rue Gaston Boissier, 75015 Paris, France; zaccaria.silvestri@lecnam.net; 6 Laboratoire National de Métrologie et d’Essais (LNE), 1 Rue Gaston Boissier, 75015 Paris, France; djilali.bentouati@lne.fr

**Keywords:** pressure standard, Fabry–Pérot refractometer, transportable, gas modulation refractometry (GAMOR), ring comparison

## Abstract

Fabry–Pérot-based refractometry has demonstrated the ability to assess gas pressure with high accuracy and has been prophesized to be able to realize the SI unit for pressure, the pascal, based on quantum calculations of the molar polarizabilities of gases. So far, the technology has mostly been limited to well-controlled laboratories. However, recently, an easy-to-use transportable refractometer has been constructed. Although its performance has previously been assessed under well-controlled laboratory conditions, to assess its ability to serve as an actually transportable system, a ring-type comparison addressing various well-characterized pressure balances in the 10–90 kPa range at several European national metrology institutes is presented in this work. It was found that the transportable refractometer is capable of being transported and swiftly set up to be operational with retained performance in a variety of environments. The system could also verify that the pressure balances used within the ring-type comparison agree with each other. These results constitute an important step toward broadening the application areas of FP-based refractometry technology and bringing it within reach of various types of stakeholders, not least within industry.

## 1. Introduction

Conventional force-per-area mechanical pressure standards, such as the dead-weight piston gauge (hereafter, for simplicity, referred to as pressure balance), have been used extensively in metrology for many decades [1,2]. For atmospheric pressures and above, their performance is still unprecedented, but for the lower pressure regime, their relative performances become gradually worse, and for pressures below a few kPa, they can often not be operated at all due to mechanical limitations [3,4,5].

A possible means to remedy such shortcomings is to employ quantum-based instrumentation that does not rely on any moving parts, and instead assesses pressure by utilizing knowledge of some fundamental properties of the gas [6]. Such instrumentation has over the last few decades experienced a significant development [7,8,9,10,11,12,13,14,15,16,17,18]. The interest in this type of techniques was further enhanced by the 2019 redefinition of the SI, in which the Boltzmann constant received an exact value [19]. This enabled the possibility for quantum-based methods to realize the pascal by primary means through an equation of state.

Although several promising quantum-based methods, with different strengths and weaknesses, are being developed, the arguably most promising one for the lower pressure regime is Fabry–Perot (FP) refractometry. FP refractometry is based on locking a laser to an FP cavity and assessing the increase in refractivity by measuring the change in frequency as a pure gas (typically N${}_{2}$ or He) fills the cavity. Using knowledge of some gas properties and utilizing the Lorentz–Lorenz equation [20], the molar density of the gas can be calculated. If the gas temperature is also known, the gas pressure in the cavity can be assessed using an equation of state [6,8].

Impressive results have been reached with FP refractometry, with claimed (*k* = 2) evaluated expanded uncertainties of [(2 mPa)${}^{2}$ + (8.8 $\times \;10^{-6}\cdot P$)${}^{2}{]}^{1/2}$ [21]; [(10 mPa)${}^{2}$ + (10 $\times \;10^{-6}\cdot P$)${}^{2}{]}^{1/2}$ [13]; and [(130 mPa)${}^{2}$ + (23 $\times \;10^{-6}\cdot P$)${}^{2}{]}^{1/2}$ [14] by the groups of the National Institute of Standards and Technology (NIST), Umeå University (UmU) in collaboration with the RISE Research Institutes of Sweden (RISE), and the National Institute of Metrology (NIM), respectively. However, a common denominator is that all these results were achieved under well-controlled laboratory conditions, with time-consuming and elaborate experimental preparations. Consequently, one can argue that much work is still required in order to fully establish the technology as a practically usable standard and as a means to assess pressure.

A crucial step for this is to demonstrate the capability of the technique to operate outside a single well-controlled environment. Motivated by this, a transportable refractometer, henceforth referred to as the transportable optical pascal (TOP), has jointly been constructed by RISE and UmU. Although the system has previously been scrutinized in terms of accuracy and precision [13,22], its ability to serve as a transportable system has not yet been assessed.

In order to demonstrate this, it was used, within the EMPIR 18SIB04 QuantumPascal project for a ring-type comparison of a set of strategically selected pressure balances at some European national metrology institutes (NMIs). The measurement campaign was initialized by a series of measurements at the RISE Research Institutes of Sweden in Borås, after which the system was shipped to, and measurements were performed at, the Physikalisch-Technische Bundesanstalt (PTB) in Berlin, the Istituto Nazionale di Ricerca Metrologica (INRiM) in Turin, and the Laboratoire National de Métrologie et d’Essais (LNE-CNAM) in Paris, before it was sent back to RISE in Borås for a concluding series of measurements.

Note though that while an ordinary ring comparison is made with the aim of assessing the performance of (or to compare) various standards, the ring-type comparison performed in this work was carried out with the main purpose of demonstrating the ability of the refractometer to be transported and swiftly set up in order to be able to assess pressures with retained performance.

This paper first gives a description of how the TOP was built to withstand harsh transportation and enable swift setup and dismantling. It then presents the measurement procedure that was used for the assessments at the various sites visited. It thereafter describes how the initial series of measurements at RISE using a pressure balance was carried out and how it was utilized as a base for a characterization of the TOP to enable successive measurements at the other sites. Finally, the paper presents the results and conclusion from the measurements, demonstrating that the system can be used as a traveling standard for assessments of pressure, as well as providing some future outlooks for improvements of the system.

## 2. Transportable Refractometer

An in-depth description of the TOP system has previously been given by Forssén et al. [22]. Hence, only a brief summary of its key properties is given here.

The TOP is constructed in a similar way to the stationary FPC-based refractometry system (denoted as the stationary optical pascal, SOP) that is based on a dual-FP-cavity (DFPC) made of Invar and makes use of the gas modulation refractometry (GAMOR) methodology, described in detail elsewhere [23,24]. One cavity acts as the measurement cavity in which gas is repeatedly filled and evacuated, while the other is used as a reference cavity that is continuously evacuated. The latter one provides a frequency reference to which the frequency changes in the measurement cavity can directly be measured by a fast photodiode as a beat frequency.

The GAMOR methodology relies on a modulation of the gas within the measurement cavity (with typical modulation cycles of hundreds of seconds) to allow for a linear interpolation between consecutive empty cavity measurements that constitutes a baseline for the assessment of refractivity [23]. As this makes the systems insensitive to linear drifts, GAMOR-based systems do not have to be extensively temperature-stabilized. This enables the realization of transportable refractometry systems with fast setup and the use of automated measurement procedures, as well as stable operation.

**Figure 1:**
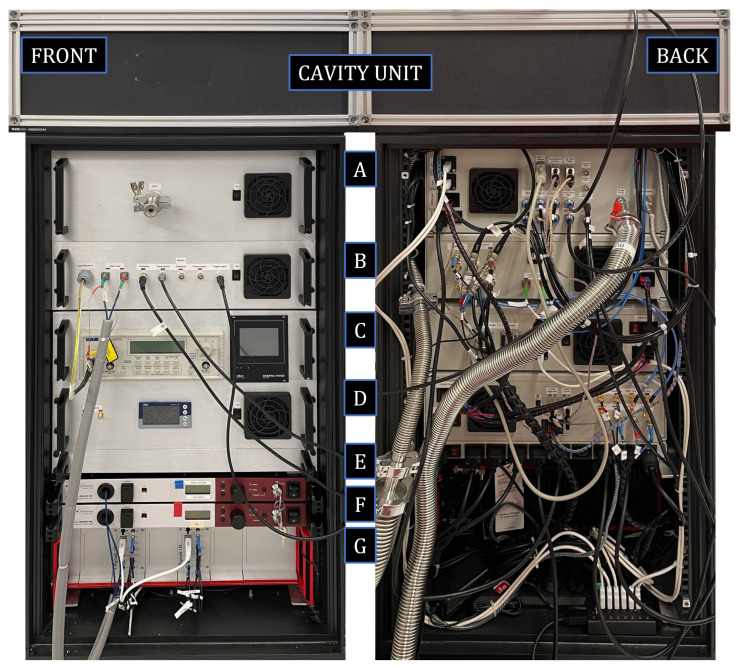
Pictures of the TOP from the front (**left**) and back (**right**). On top of the rack sits a temperature-regulated aluminum breadboard with an isolating enclosure, within which the DFPC is located. The rack contains seven modules, denoted A–G. (A) The gas inlet system consisting of a mass flow controller and an electronic pressure controller. (B) Optics, passive fiber optical components (e.g., circulators and isolators), and opto-electronics (EOMs and AOMs). (C) Frequency counter and vacuum gauge controllers. (D) Power supplies and control unit for the heating. (E) A 230 V power distribution unit. (F) Two Er-doped fiber lasers. (G) Two digital locking modules. Reproduced with permission from Forssén et al. [22].

To simplify the transportation and initialization, two main design compromises were made with respect to the SOP system, viz.:

1. The system was designed to fit in a 120 cm high wheel-equipped 19-inch rack. This is obviously non-ideal in terms of stability; it would be preferable to place the system on a firm and stable surface, such as a rigid optical table. However, this overall design has the advantages that it makes it easy to move the refractometer within laboratories and, at the same time, minimizes the footprint of the system, which otherwise can be a problem at some visited laboratories. A picture of the front and back of the system is shown in Figure 1.
2. The TOP assesses the temperature of the cavity by using two Pt-100 sensors whose outputs are assessed by a data acquisition (DAQ) system. This is in contrast to the stationary system that uses a thermocouple directly referred to an active gallium fixed-point cell. The reason for this downgrade in terms of accuracy is that the gallium cell adds unwanted complexity to the system that most notably increases the setup time and limits the measurement to certain time windows when the gallium is in its proper melting state. To ensure traceability, without the gallium fixed-point, an external temperature measurement device (Hart 1502A) and an accompanying calibrated Pt-100 probe was brought as hand luggage to each visit and used to calibrate the Pt-100 probes and the DAQ system of the TOP prior to each series of measurements. To evaluate the stability of the temperature assessment module, it was calibrated at RISE both before and after the measurement campaign. The discrepancy between these two calibrations was below the resolution of the instrument (1 mK), providing an estimated uncertainty limited by the resolution of the instrument of 2 ppm (see below).

The TOP can, in principle, operate as a primary standard, as was demonstrated in 2021 when its (*k* = 2) expanded uncertainty was assessed to [(16 mPa)${}^{2}$ + (28 $\times \;10^{-6}\cdot P$)${}^{2}{]}^{1/2}$ [13]. However, because of a multitude of reasons, primarily the following ones: (i) the aforementioned characterization was performed only in the 10–30 kPa range, (ii) the FP cavity had been refurbished since the characterization (the mirrors had been dismounted and cleaned), which presumably could have affected the cavity distortion, (iii) it had not been verified that the characterization of the system would not be adversely affected by the transportation from UmU to RISE, (iv) a new characterization was considered to be too time-consuming and complex to incorporate before the measurement campaign was to be performed (it had to be performed within the finite time frame of an ongoing EMPIR project), and (v) the main aim of this work was to assess the ability of the TOP instrumentation to withstand harsh transportation and operate outside a well-stabilized laboratory environment, it was decided that the instrumentation should be operated as an intermediate device, analogous to a transfer standard.

Finally, in order to enable the easy, rapid, and cost-efficient transportation of the TOP, it was designed so that its total footprint, including all accompanying external equipment (such as vacuum equipment and pumps), is smaller than a standard EUR pallet (1200 × 800 mm). Due to this, standard shipping services could be used to transport the system between the various NMIs. During the transportation, the system was subjected to rough handling (evidenced by visible damage to the exterior of the parcel) [25], making the transportation indirectly serve as a stress test. As is further discussed below, despite this, the performance of the TOP was virtually unchanged throughout the measurement campaign.

## 3. Measurement Procedure

### 3.1. Establishment of Measurement Procedures

To ensure comparable conditions for the assessments of pressure at all visited sites, a measurement procedure was established before the initiation of the measurement campaign. This stipulated that each site visit should be carried out within a working week. In order to achieve this, the following agenda was set.

At each site, nitrogen should be supplied by canisters (with at least 5 N purity) and assessments of nine pressures (ranging from 10 to 90 kPa with steps of 10 kPa) should preferably be carried out within one working day. This implied that roughly one hour was to be spent per pressure level. Since GAMOR cycles of 300 s were used (comprising 150 s for the filling and stabilization of the gas and 150 s for evacuation), and since the allocated hour also needed to incorporate time for changing nominal pressure and stabilization of the pressure balance, primarily stabilization of the temperature and pumping of the bell jar, it was decided that three GAMOR cycles should be made at each pressure. It is worth noting that, although more cycles at each pressure level could in theory improve the statistics, three points at each pressure level, which allowed all measurements to be performed within one working day, was considered sufficient to demonstrate the stability of the TOP and the repeatability of the measurements.

### 3.2. Day One—Unpacking, Setup, and Installation

Given that the system had been successfully packed and shipped beforehand, the first day was allocated to traveling to the host site by RISE/UmU personnel, as well as the unpacking, setup, and installation of the TOP (which normally takes about two hours). When this had been carried out, the system was left to thermally stabilize overnight.

### 3.3. Day Two—Optical Alignment, Control/Verification, and Stabilization

Day two was allocated to optical alignment and control/verification of the functionality of the system. As a part of this, all critical parameters, primarily the alignment of the laser beams through the cavities, the free spectral range of the cavity, the wavelength of the lasers, and the calibration of the temperature probes, were addressed and, whenever appropriate, assessed. After this, the system was left to stabilize further until the next day.

### 3.4. Day Three—Assessments

Day three constituted the day when all measurements were performed. The nine set pressures were measured in a randomized order. The TOP was controlled by the RISE/UmU personnel while the pressure balance was run by a person associated with the visited site. For each pressure measurement, the gas-handling system of the TOP was first used to roughly set the pressure to the nominal set pressure of the pressure balance. The operator of the pressure balance was thereafter given about 50 s to fine-tune the height and rotational speed of the piston to the operational standard of the pressure balance, i.e., to set a known pressure. When a set pressure had been reached, a refractivity assessment with gas in the (measurement) cavity, as required for the GAMOR measurement methodology, was performed.

### 3.5. Day Four—Spare Day and Packing

Day four was considered to be a spare day, in practice allowing for additional measurements in case any unforeseen problems would have arisen during day three. At the end of day four, however, the system was to be fully packed and ready for shipment to the next site (which roughly took two hours).

### 3.6. Day Five—Spare Day for Packing

In case the available time for any spare activity during day four would have been insufficient, the protocol stipulated that, depending on the travel arrangements of the visiting personnel, the morning of day five could alternatively be utilized to pack the TOP. However, it turned out that this alternative was not utilized at any of the visited sites. Day five was therefore solely used for the return journey for the visiting personnel from RISE/UmU.

### 3.7. Packing—Preparation for Shipping

The shipment of the packed TOP to the next site was administrated by the visited site during the week(s) that followed. In all cases except one, the transportation of the TOP was carried out by a commercial shipping company by truck; for the transportation from LNE-CNAM back to RISE, it was transported by flight.

## 4. Measurements and Results

### 4.1. Initial Measurement and Characterization

The measurement campaign was initiated by a series of measurements at RISE on a pressure standard (Ruska 2465A-754) against which the TOP was characterized (referred to as RISE1). The procedure for the characterization is described in detail by Forssén et al. [25]. In short, the TOP measured the pressure generated by the pressure standard in the 10–90 kPa range, which was the largest overlapping working range of all pressure standards addressed in the ring comparison and the TOP (by its construction, the TOP is currently limited to pressures below 100 kPa).

In order to obtain a continuous characterization, a second-order polynomial of the form $a+bP_{PB}+cP_{PB}^{2}$, where *a*, *b*, and *c* are fitting parameters and $P_{PB}$ is the pressure set by the pressure balance, henceforth denoted as $P_{TOP}^{fit}$, was fitted to the data taken by the TOP and evaluated by the standard expression for refractivity [26] with the deformation parameter set to zero (denoted as $P_{TOP}$).

**Figure 2:**
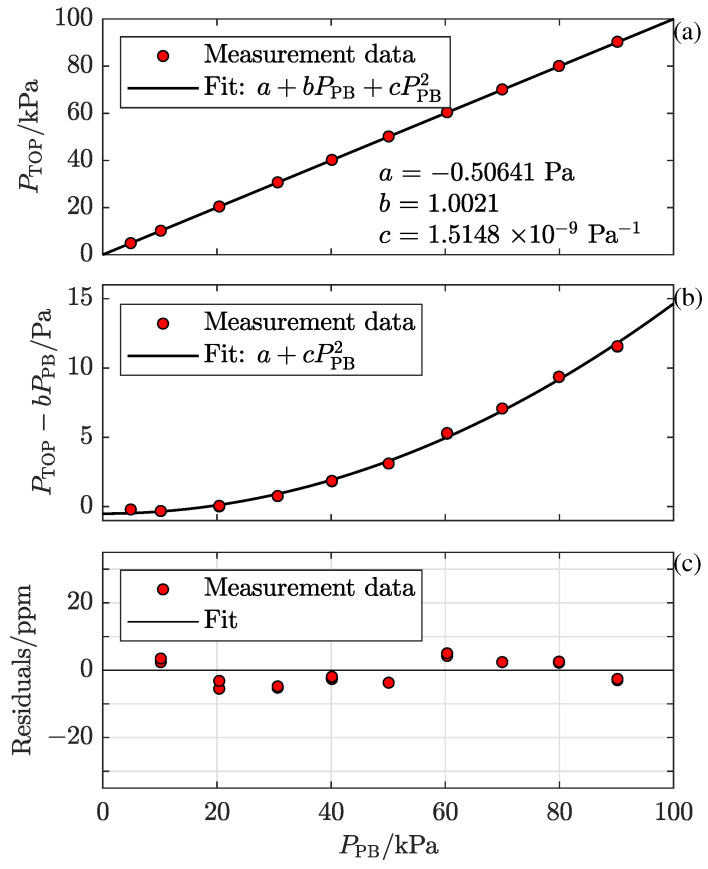
The first measurement series at RISE (RISE1). The red markers represent measurement data points taken by the TOP, and the solid lines and curves represent polynomial fits to them, all as a function of the pressure set by the pressure balance, $P_{PB}$. Panel (**a**) shows, by the individual markers, the pressure assessed by the TOP evaluated by the standard expression for refractivity with the deformation parameter set to zero, $P_{TOP}$, in kPa. The solid curve represents the fit, $P_{TOP}^{fit}$. Panel (**b**) displays the non-linear components of panel (**a**) given by, for the individual data markers, $P_{TOP}-bP_{PB}$ and, for the fit, $a+cP_{PB}^{2}$, respectively, in Pa. Panel (**c**) illustrates the residuals of the fit from panel (**a**,**b**) in relative units, i.e., $\left(P_{TOP}-P_{TOP}^{fit}\right)/P_{PB}$, in parts per million (ppm), which also represent the relative deviations in the pressure assessed by the characterized TOP, $P_{TOP}^{Ch}$, from the pressure set by the pressure balance, $P_{PB}$.

In Figure 2a, the individual measurement data points ($P_{TOP}$) are shown as red solid markers while the fit ($P_{TOP}^{fit}$), where a=−0.50641 Pa, b=1.0021, and $c=1.5148\;\times \;10^{-9}$ Pa${}^{-1}$, is shown by the solid black curve.

As was discussed by Forssén et al. [25], the deviation in the *b* parameter from unity can be mainly attributed to the pressure-induced cavity deformation. The non-linearity (given by the $cP_{PB}^{2}$ term) can be attributed to a second-order pressure dependence of the deformation. To show the contribution from the non-linear response to the signal, the data points and the second-order fit are shown in Figure 2b without the dominating linear part of the fit (i.e., as $P_{TOP}-bP_{PB}$ and $a+cP_{PB}^{2}$, respectively).

The residuals of the fit in relative units, represented by $\left(P_{TOP}-P_{TOP}^{fit}\right)/P_{PB}$, are shown in parts per million (ppm) in Figure 2c. Under the condition that the response of the refractometer does not have any higher-order non-linearities than that given by the second-order polynomial above, which is a reasonable assumption, the characterized response of the refractometer, $P_{TOP}^{Ch}$, which is given by the solution of the $P_{TOP}=a+bP_{TOP}^{Ch}+c{\left(P_{TOP}^{Ch}\right)}^{2}$ equation, is, except for measurement fluctuations, given by $P_{PB}$. This implies that the residuals of the fit also represent the relative deviations in the pressure assessed by the characterized TOP, $P_{TOP}^{Ch}$, from the pressure set by the pressure balance, $P_{PB}$.

### 4.2. The Ring Comparison

**Figure 3:**
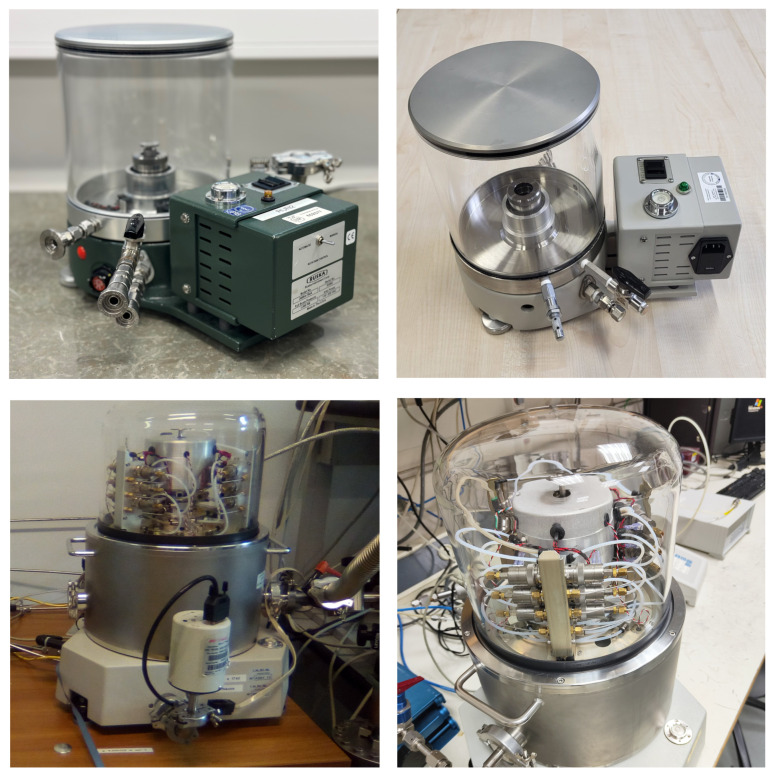
Pictures of the four different pressure balances used in the ring comparison. (**Top left**): RISE Ruska 2465A-754, (**top right**): PTB Fluke 2465A-754, (**bottom left**): INRiM DHI-Fluke PG7601, (**bottom right**): LNE DHI-Fluke PG7607.

The ring comparison campaign comprised four different pressure balances, a Ruska 2465A-754 at RISE, a Fluke 2465A-754 at PTB, a DHI-Fluke PG7601 at INRiM, and a DHI-Fluke PG7607 at LNE, as shown in Figure 3.

Table 1 summarizes, in chronological order, the five measurement series of the campaign (each series has been denoted by which NMI it was performed at) and includes the date of the measurement, the model of the pressure balance, and its (*k* = 2) expanded uncertainty. It is worth noting that the individual measurement locations were not chosen based on the performance of the pressure balances addressed (e.g., PTB has pressure balances with lower uncertainties at other laboratories); the choices were instead based on availability, convenience, cost, and environmental aspects as the traveling in some cases could be combined with meetings and other joint activities.

As previously mentioned, three individual measurements were performed at each pressure. Each measurement was thoroughly evaluated and a few individual points with confirmed problems, attributed to either unreliable conditions in the pressure balance or the TOP, defined as outliers, were removed. (The points removed were attributed to either of two causes: jamming in the piston–cylinder assembly, which was identified by the operator during the measurements, or a loss of laser lock that coincided with the measurement window, observed during post-measurement analysis.)

**Figure 4:**
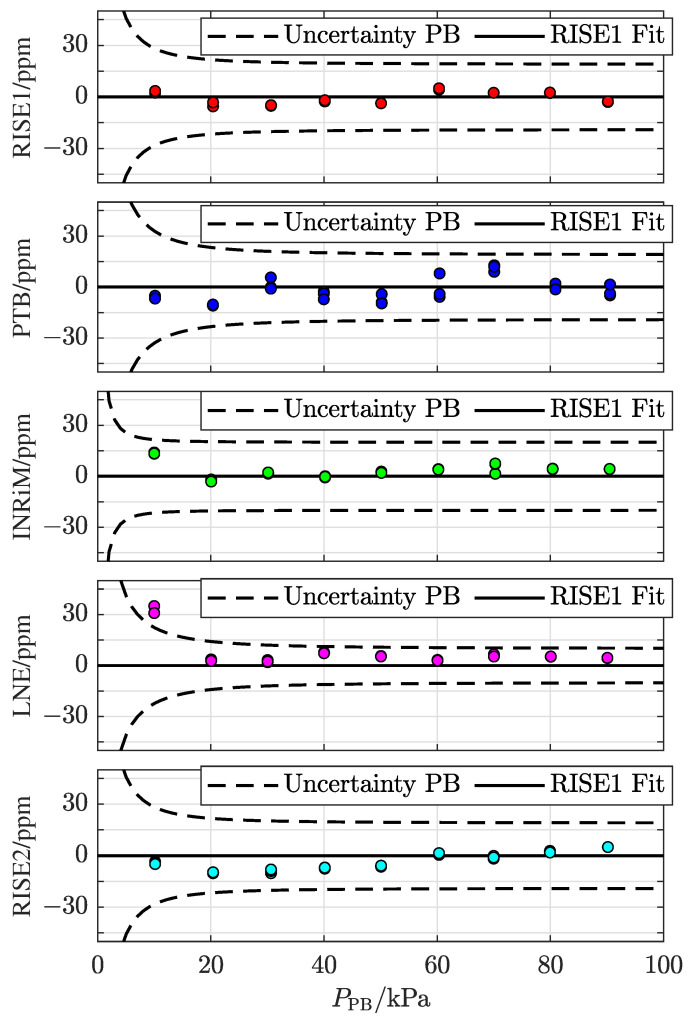
Colored circles: deviations between the pressures assessed by the TOP refractometer, $P_{TOP}^{Ch}$, and the set pressures of the pressure balances, $P_{PB}^{i}$, from the measurements performed at the various NMIs (i.e., with *i* being RISE1, PTB, INRiM, LNE, and RISE2, respectively). Black horizontal lines: polynomial fits of the initial characterization. The dashed curves represent the uncertainty values for the pressure balance used at the corresponding NMI. The first panel, denoted as RISE1, contains the same data as in Figure 2c.

Figure 4 presents a compilation of the five measurement series, given as the difference between the pressure assessed by the TOP in its characterized mode of operation, $P_{TOP}^{Ch}$, and the pressure set by the pressure balance, $P_{PB}$, (in ppm), indicated by a colored circle for each measurement that, in several cases, are visually fully overlapping. (The small spread among the individual measurements taken at a given pressure and site (in most cases smaller than the size of the markers in Figure 4) is in line with the findings that the TOP has an outstanding (sub-ppm) short-term precision [22], orders of magnitude better than its uncertainty, which predominantly is given by the uncertainty in the molar polarizability of nitrogen at the wavelength and temperature utilized [13].) For comparison, in each panel, the dashed curves represent the uncertainty values for the pressure balances addressed at the various NMIs.

The measurements demonstrate that the TOP is fully capable of assessing the pressures set by the various pressure balances at the sites visited. The first data set (RISE1) is the same as in Figure 2c and is hence centered around zero per definition. The fact that also the four following data sets are all well centered around zero (and in particular the last one (RISE2)) indicates that the TOP retains its performance throughout the measurement campaign. The data also verify, as is expected, that all pressure balances agree with each other. (For the lowest pressure level at LNE-CNAM (10 kPa), there was noticeable jamming despite repeated measurement tries. Therefore, the corresponding measurement points should be associated with a larger uncertainty than the other measurements at the same institute).

**Figure 5:**
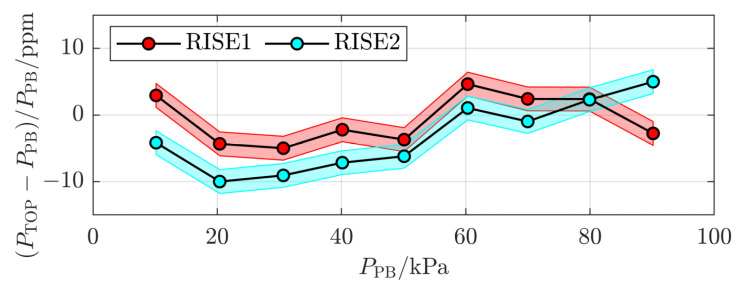
The two measurement series from RISE (RISE1 and RISE2, respectively). The shaded areas indicate the (*k* = 2) expanded uncertainty originating from the finite resolution of the temperature-measuring instrument.

To compare the results between the initial and final measurements in some more detail, Figure 5 shows the initial and final measurements at RISE (RISE1 with red markers and RISE2 with cyan markers, respectively). It can be concluded that the difference between the two measurement series is at most 8 ppm, and on average 4 ppm.

This difference can be attributed to a number of causes, predominately the finite resolution of the temperature-measuring instrumentation, changes in the leaks and outgassing rates, alterations in the pressure-induced cavity deformation, and, since the pressure balance at RISE was moved between the two series of measurements, a difference in the tilt in the pressure balance or in the bell jar residual pressure.

At the time of the ring comparison, there was no possibility to assess the individual influence of each of these causes. However, the influence of the finite resolution of the temperature-measuring instrumentation could be estimated by the use of $R_{i}/\sqrt{12}$, where $R_{i}$ is the resolution of the instrument [27], to 1 mK. Hence, the expanded uncertainty in pressure (*k* = 2) due to this, illustrated by the red and cyan shaded areas in Figure 5, could be estimated to 2 ppm. It is strictly not possible to assess any upper limit for any of the other causes. However, the discrepancy data indicate that it is unlikely that any of them, including any possible shift in the pressure-induced deformation of the cavity, is significantly above the mean deviation of 4 ppm.

## 5. Conclusions

To assess the ability of a previously constructed transportable FP-based refractometer, based on a DFPC made of Invar and utilizing the GAMOR methodology, referred to as the transportable optical pascal (TOP), this paper presents the results from a ring-type comparison in which it was used to assess the pressures set by four traceable pressure balances located at four European NMIs.

Since a prerequisite for performing a quantitative evaluation of key comparison data, such as key comparison reference values (KCRVs) and degrees of equivalences (DOEs), is “a well-characterized travelling standard having good short-term stability and stability during transport” [28], and since FP-based refractometers in general require extraordinary stable conditions to operate, rather than performing a quantitative evaluation of the pressure balances addressed, the ring-type comparison carried out in this work was performed with the main purpose of demonstrating the ability of the TOP to undertake such a comparison, in particular regarding its stability.

It was found that the difference between the pressures assessed by the refractometer and those set by the various pressure balances are largely centered around zero, which indicates that the TOP retains its performance throughout the measurement campaign. The results also show, as is expected, that the assessed pressures of the pressure balances agree with the set pressures within their uncertainties. It was also found that the difference between the initial and final measurements at RISE (RISE1 and RISE2, respectively) was significantly smaller than the uncertainties of the pressure balances: at most 8 ppm, and on average 4 ppm. This demonstrates the capability of the TOP refractometer to function as a transportable system; it shows, in particular, that it can be transported and swiftly set up to be operational in different environments with retained performance. To the knowledge of the authors, this is the first time an FP-based refractometer has been transported between various NMIs and demonstrated functionality with preserved performance.

The results also give important information about possible means to improve on the performance of the refractometer. Although only the amount of uncertainty attributed to the finite resolution of the temperature-measuring instrumentation could be estimated (2 ppm), it is possible that also changes in the leaks and outgassing rates and potential alterations in the pressure-induced cavity deformation contribute to the uncertainty. A redesign of the temperature control and assessment units will not only improve the resolution, but also, by upgrading the control unit, the time to achieve thermal stability can be significantly reduced (from 1–2 days to a few hours), allowing measurements to be performed almost directly after installation. The effect from leaks and outgassing can be reduced by improving parts of the vacuum system, most importantly some of the connectors and valves. Due to the modular design of the TOP, if any particular cause is identified as the major contributor to the uncertainty, it is easy to upgrade the system with respect to that particular cause.

Furthermore, the results also indicate that it is feasible in the future, following an adequate characterization of the system (primarily an assessment of the pressure-induced cavity deformation) and a general upgrade to the system (including most notably a higher accuracy temperature assessment), to operate the system as a transportable primary standard with a performance limited by the knowledge of molar gas constants.

This work thereby paves the way for future measurement campaigns addressing both different types of conventional low-uncertainty standards for gas pressures and novel emerging quantum-based methods that are currently under development.

Finally, this work is also an important step toward bringing the technology of quantum-based methods for the assessment and realization of pressure within the reach of stakeholders and industry, for a variety of applications.

## Figures and Tables

**Table 1:** Summary of the five measurement series. For simplicity, the expanded uncertainties (*k* = 2) are given with a simplified notation in the format A+B·P , which, in reality, corresponds to ${\left[A^{2}+{\left(B\cdot P\right)}^{2}\right]}^{1/2}$, where *A* represents the pressure-independent contribution to the uncertainty while *B* is the coefficient in the pressure-dependent contribution.

**RISE1: Borås, Sweden**	Pressure Balance	Ruska 2465A-754
**Date:** 19 January 2022	Uncertainty (*k* = 2)	0.21 Pa + $19\;\times \;10^{-6}P$
**PTB: Berlin, Germany**	Pressure Balance	Fluke 2465A-754
**Date:** 17 February 2022	Uncertainty (*k* = 2)	0.27 Pa + $19\;\times \;10^{-6}P$
**INRiM: Turin, Italy**	Pressure Balance	DHI-Fluke PG7601
**Date:** 6 April 2022	Uncertainty (*k* = 2)	0.08 Pa + $20\;\times \;10^{-6}P$
**LNE: Paris, France**	Pressure Balance	DHI-Fluke PG7607
**Date:** 21 June 2022	Uncertainty (*k* = 2)	0.20 Pa + $10\;\times \;10^{-6}P$
**RISE2: Borås, Sweden**	Pressure Balance	Ruska 2465A-754
**Date:** 10 October 2022	Uncertainty (*k* = 2)	0.21 Pa + $19\;\times \;10^{-6}P$

## Data Availability

Data are contained within the article.

## Abbreviations

The following abbreviations are used in this manuscript:

CNAM	Conservatoire National des Arts et Métiers
DAQ	Data acquisition system
DFPC	Dual-Fabry–Pérot cavity
DOEs	Degrees of equivalences
EMPIR	European Metrology Programme for Innovation and Research
FP	Fabry–Pérot
GAMOR	Gas modulation refractometry
INRiM	Istituto Nazionale di Ricerca Metrologica
KCRVs	Key comparison reference values
LNE-CNAM	Laboratoire Commun de Métrologie (CNAM)
NIM	National Institute of Metrology
NIST	National Institute of Standards and Technology
NMI	National Measurement Institute
ppm	parts per million
PTB	Physikalisch-Technische Bundesanstalt
RISE	Research Institutes of Sweden
SOP	Stationary optical pascal
TOP	Transportable optical pascal
UmU	Umeå University
